# Hexa-*peri*-benzocoronene with two extra K-regions in an *ortho*-configuration†

**DOI:** 10.1039/d0sc04649c

**Published:** 2020-10-23

**Authors:** Tim Dumslaff, Yanwei Gu, Giuseppe M. Paternò, Zijie Qiu, Ali Maghsoumi, Matteo Tommasini, Xinliang Feng, Francesco Scotognella, Akimitsu Narita, Klaus Müllen

**Affiliations:** Max Planck Institute for Polymer Research Ackermannweg 10 55128 Mainz Germany qiu@mpip-mainz.mpg.de muellen@mpip-mainz.mpg.de; Istituto Italiano di Tecnologia, Center for Nano Science and Technology Milano 20133 Italy francesco.scotognella@polimi.it; Dipartimento di Chimica, Materiali e Ingegneria Chimica – Politecnico di Milano Piazza Leonardo da Vinci 32-20133 Milano Italy; Center for Advancing Electronics Dresden (CFAED), Department of Chemistry and Food Chemistry, Dresden University of Technology Walther-Hempel-Bau Mommsenstrasse 4 01062 Dresden Germany

## Abstract

There are three possible isomers for hexa-*peri*-hexabenzocoronene (HBC) with two extra K-regions, but only two of them have been reported, namely with the *meta*- and *para*-configurations. Herein, we describe the synthesis of HBC **4** with two extra K-regions in the *ortho*-configuration, forming a longer zigzag edge compared with the other two isomers. The structure of **4** was validated by laser desorption/ionization time-of-flight mass analysis and nuclear magnetic resonance spectra, as well as Raman and infrared spectroscopies supported by density functional theory calculations. The optical properties of **4** were investigated by UV/vis absorption and ultrafast transient absorption spectroscopy. Together with the analysis of aromaticity, the influence of the zigzag edge on the π-conjugation pathway and HOMO–LUMO gaps of the three isomers were investigated.

## Abstract 2

We reported the synthesis of hexa-*peri*-benzocoronene (HBC) with two extra K-regions adopting an *ortho*-configuration. The systematical study provides deep insights about the effect of zigzag edge on the π-conjugated pathway and molecular design.

## Graphical abstract

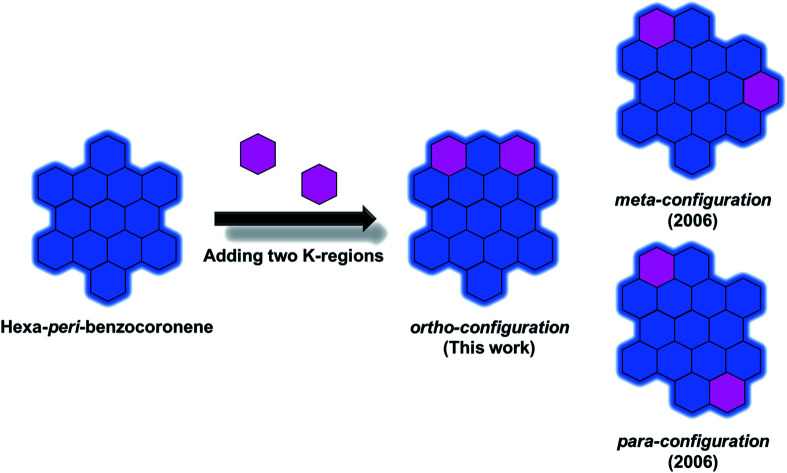

## Introduction

**Fig. 1:**
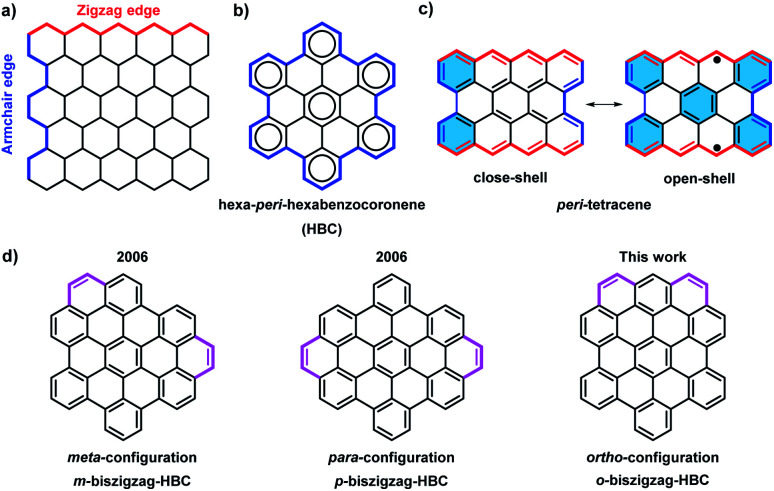
(a) Illustration of the armchair and zigzag structures in PAHs; (b) all-benzenoid structure of HBC; (c) structure of *peri*-tetracene; (d) three isomers for HBC with two extra K-regions.

Over the past century, there has been a rapid development in the field of polycyclic aromatic hydrocarbons (PAHs) with main interests ranging from organic chemistry to materials science. Various PAHs have been applied in organic field-effect transistors (OFETs), dye-sensitized solar cells, and organic light-emitting diodes (OLEDs).^1^ Through intensive experimental and theoretical studies, the optical properties, chemical reactivity, and aromaticity of PAHs were found to strongly depend on their sizes, shapes, and edge structures.^2^ Armchair and zigzag are the two most representative edge structures of PAHs (Fig. 1a). According to Clar's aromatic sextets rule,^3^ PAHs with solely armchair edges, such as hexa-*peri*-hexabenzocoronene (HBC), can be drawn as fully benzenoid structures without additional double bonds (Fig. 1b), and display high stability and larger gaps between the highest occupied molecular orbital (HOMO) and the lowest unoccupied molecular orbital (LUMO).^4^ On the contrary, PAHs with zigzag edges, such as triangle-^5^ and rhombus-shaped PAHs,^6^ as well as *peri*-acene-derivatives,^7^ are not fully benzenoid and typically show smaller HOMO–LUMO gaps as well as diverse spin states. Moreover, those cases with longer zigzag edges such as *peri*-tetracene display lower stability and open-shell biradical character due to the formation of additional aromatic sextet rings in the open-shell form (Fig. 1c).^7*d*,*e*^

With the introduction of C2 units at the bay regions, the fully benzenoid HBC can be transformed into not fully benzenoid derivatives with extra K-regions, forming zigzag edge peripheries. HBCs with one to four K-regions have thus far been synthesized,^8^ exhibiting substantially different optoelectronic properties. A higher number of extra K-regions generally leads to smaller energy gaps. For HBCs with two extra K-regions, namely so-called biszigzag-HBCs, there are three possible isomers with *ortho*-, *meta*-, and *para*-configuration of the K-regions (Fig. 1d). Until now, **m-biszigzag-HBC** and **p-biszigzag-HBC** have been reported in 2006,^8*c*^ but **o-biszigzag-HBC** has remained elusive for a long time.

Herein, we report an efficient synthesis of **o-biszigzag-HBC** derivative **4** from a benzotetraphene-based “U-shaped” precursor **3**, having a preinstalled zigzag edge. The successful formation of **4** was validated by matrix-assisted laser desorption/ionization time-of-flight mass spectrometry (MALDI-TOF MS) and nuclear magnetic resonance (NMR) spectra, as well as infrared (IR) and Raman spectroscopies supported by density functional theory (DFT) simulations. The photophysical properties of **4** were investigated by UV/vis absorption and ultrafast transient absorption spectroscopies, which revealed an interplay of stimulated emission and photoinduced absorption. Moreover, the HOMO–LUMO gaps and aromaticity of **o-biszigzag-HBC**, **m-biszigzag-HBC**, and **p-biszigzag-HBC** were studied by DFT calculations, providing an insight into the effect of the additional two K-regions on the HBC core with different configurations.

## Results and discussion

### Synthesis

**Scheme 1:**
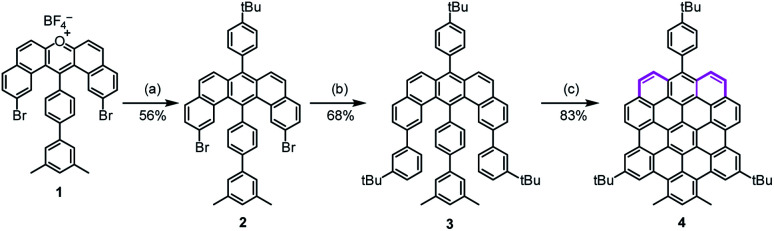
Synthetic route towards **4**. Reagents and conditions: (a) sodium 2-(4-*tert*-butylphenyl)acetate, benzoic anhydride, 150 °C, 24 h, 56%; (b) 3-*tert*-butylphenylboronic acid, Pd(PPh_3_)_4_, K_2_CO_3_, H_2_O, ethanol, toluene, 110 °C, 24 h, 68%; (c) DDQ/TfOH, dichloromethane, room temperature, 8 h, 83%. DDQ: 2,3-dichloro-5,6-dicyano-1,4-benzoquinone, TfOH: trifluoromethanesulfonic acid.

In the synthesis of **p-biszigzag-HBC** and **m-biszigzag-HBC**, the K-regions were derived from precursors containing phenanthrene units.^8*c*^ However, **o-biszigzag-HBC** cannot be synthesized following the same strategy due to the absence of two separated phenanthrene units. Therefore, we envisaged the synthesis of **o-biszigzag-HBC** derivative **4** through the oxidative cyclodehydrogenation of U-shaped precursor **3** (Scheme 1), in analogy to a previous synthesis of tetrazigzag-HBC.^8*a*^ Adapting our established procedure, nucleophilic oxygen/carbon exchange of pyrylium salt **1** led to the U-shaped compound **2**.^9^ Subsequently, Suzuki coupling of **2** provided the key precursor molecule **3** with two K-regions in an *ortho*-configuration. The final oxidative cyclodehydrogenation of **3** with 2,3-dichloro-5,6-dicyano-1,4-benzoquinone (DDQ) and trifluoromethanesulfonic acid (TfOH) afforded **4** in a satisfying yield of 83%.

### Structural characterizations

**Fig. 2:**
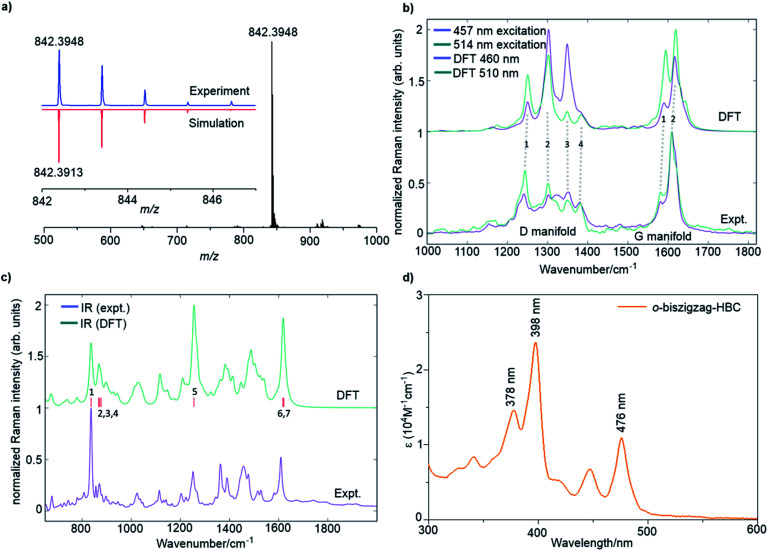
(a) MALDI-TOF MS of **4**. Inset: isotopic distribution compared with the mass spectrum simulated for C_66_H_50_. (b) FT-Raman spectra of **4** compared with results from DFT calculations over the D and G regions. (c) Micro FT-IR spectrum of **4** and the simulated spectrum by DFT calculations. (d) UV/vis absorption spectrum **4** in THF. Concentration: 2 × 10^−5^ M.

A combination of the MALDI-TOF MS, NMR, IR, and Raman spectroscopy offered clear evidence for the successful formation of **4**. As shown in Fig. 2a, the MALDI-TOF MS of **4** displayed an intense signal at *m*/*z* = 842.3948, fully consistent with its calculated molecular mass of 842.3913. Furthermore, the observed isotopic distribution was in perfect agreement with the simulated pattern (red line) based on the elemental composition of C_66_H_50_. ^1^H and ^13^C NMR spectra of **4** could be recorded in a mixture of tetrahydrofuran-*d*_8_ and CS_2_ (2 : 1) at room temperature, corroborating successful formation of **4** (Fig. S11 and S12†). The ^1^H NMR shows three clearly resolved singlet peaks in the aromatic region, which could be assigned with the assistance of 2D correlation spectroscopy (COSY) and nuclear Overhauser effect spectroscopy (NOESY) NMR spectra (Fig. S13 and S14†). However, the other doublet signals could not be unambiguously assigned because the low solubility of **4** hindered the acquisition of appropriate 2D NMR spectra.

The Raman analysis was performed on a powder sample of **4** with excitation at 457 and 514 nm, and the spectra were compared with the simulated ones by DFT calculations (Fig. 2b). To our delight, the experimental Raman spectra were in accordance with the DFT-calculated results, thus supporting the synthesis of **4**. Notably, the spectra displayed several components in the D (∼1300 cm^−1^) and G regions (∼1600 cm^−1^). In particular, the observed D peaks could be assigned to four ring-vibration modes of the skeleton as computed by DFT (Fig. S4†). A doublet was observed in the G band, which was also consistent with the two most intense G peak ring vibration modes computed by DFT (Fig. S5†).

The IR-spectrum of a powder sample of **4** was measured in a diamond anvil cell and also compared with the spectrum obtained by DFT calculations (Fig. 2c and S6†). Notably, the experimental IR spectrum was again fully consistent with the calculated signals. It is remarkable to note that the collective out-of-plane C–H bending of **4** can be observed at 837 cm^−1^ (854 cm^−1^, unscaled DFT mode 1). The weaker bands observed at 856, 871, and 877 cm^−1^ can be assigned to modes 2, 3, and 4 computed by DFT at 885, 889, and 895 cm^−1^, respectively (unscaled wavenumbers). The IR modes 2 and 3 are ascribed to C–H out-of-plane vibrations located on the edge of the molecule that is opposite to the zigzag side, whereas mode 4 is a collective in-plane deformation of the aromatic backbone. The aryl group attached to the zigzag edge displays a C–H out-of-plane bending that falls within the band observed at 837 cm^−1^ (such mode is computed at 852 cm^−1^ by DFT – unscaled). The in-plane aromatic C–H bending of the dimethyl substituted benzene rings feature a medium IR-absorption band observed at 1252 cm^−1^ (1281 cm^−1^, unscaled DFT mode 5). Finally, the strong band observed at 1608 cm^−1^ can be assigned to ring stretching vibrations with nuclear displacement patterns similar to those found for the Raman G modes, although such modes are different from the Raman active ones. The calculation details can be found in the ESI.†

The optical properties of **4** were investigated by UV/vis absorption spectroscopy in tetrahydrofuran (THF) (Fig. 2d) as well as the time-dependent DFT (TDDFT) calculations (Fig. S1 and Table S1†). Three absorption bands were observed at 476 nm (2.61 eV), 398 nm (3.12 eV), and 378 nm (3.28 eV). Correspondingly, the TDDFT calculations on **4** showed three optically allowed vertical transitions with large oscillator strengths at 488 nm (2.54 eV, p-band), 415 nm (2.99 eV, β-band), and 405 nm (3.06 eV, β′-band) as presented in Fig. S1a.† Here, p-, β-, and β′-bands are based on Clar's notation and mainly correspond to HOMO → LUMO, HOMO → LUMO+1, and HOMO−1 → LUMO+1 transitions, respectively, which are consistent with electronic transitions based on the TDDFT calculations (Table S1†).^8*c*,10^ The α-band (HOMO−1 → LUMO), which is normally expected for PAHs, cannot be found in the recorded UV/vis spectrum because of its weak oscillator strength (0.0183) as well as the overlap with the strong p-band, which is split into three peaks by vibronic coupling (Fig. 2d) similar to our previous observation for the tetrazigzag HBC.^8*a*^

The HOMO and LUMO energy levels of **4** were calculated to be −4.75 and −1.88 eV, respectively, by DFT with a HOMO–LUMO gap of 2.87 eV (Fig. S2†). As expected from the influence of the zigzag edge, the HOMO–LUMO gap of **4** is lower than that of the parent HBC by 0.68 eV. For comparison, the pristine structure of **4** without substitutions, namely **o-biszigzag-HBC**, was also calculated to possess its HOMO at −4.94 eV and LUMO at −2.02 eV with a HOMO–LUMO gap of 2.92 eV. These were slightly different from the energy levels of **4** (Fig. S3†). Among the HOMO–LUMO gap calculations of three isomers (Fig. S3†), **p-biszigzag-HBC** exhibits the lowest energy gap (2.82 eV) compared with **o-biszigzag-HBC** (2.92 eV) and **m-biszigzag-HBC** (2.94 eV).

**Fig. 3:**
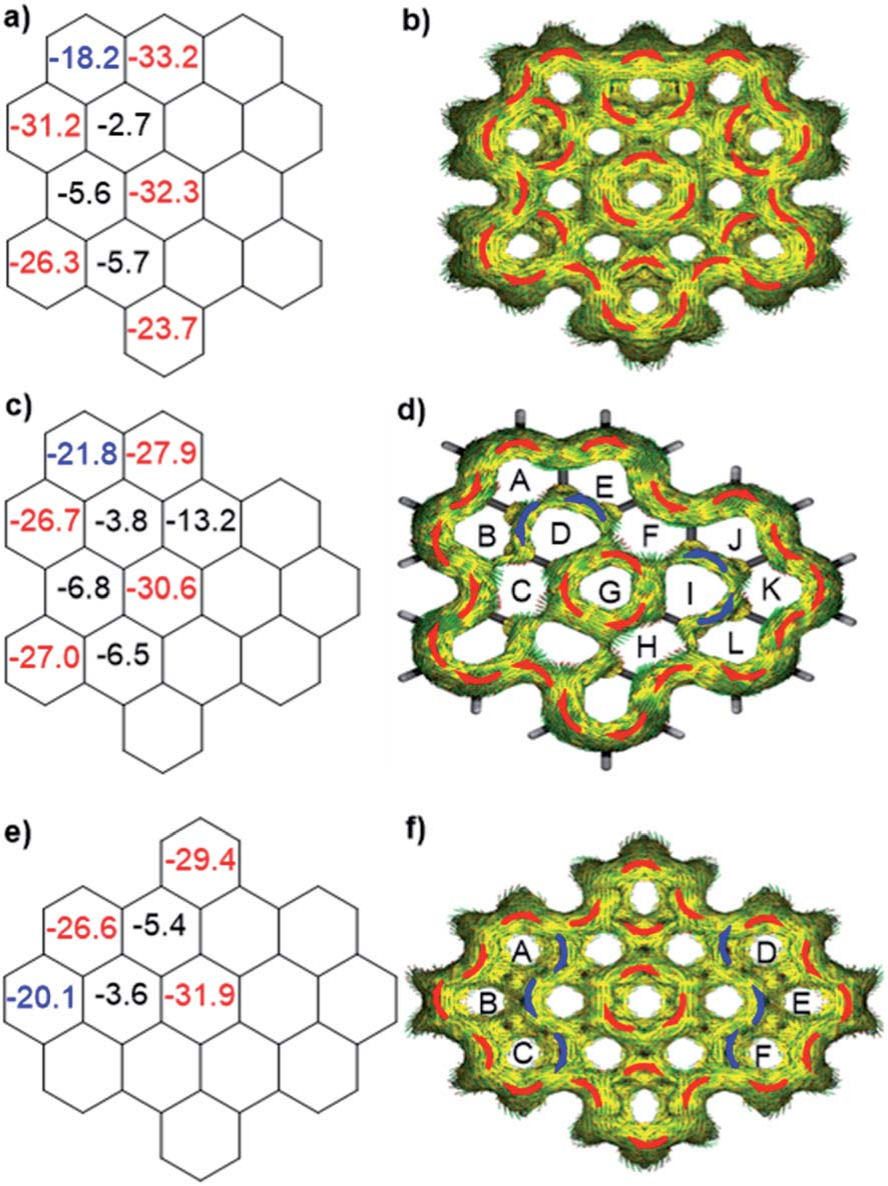
Calculated NICS(1)_*zz*_ values and ACID plots (contribution from π electrons only) of **o-biszigzag-HBC** (a and b), **m-biszigzag-HBC** (c and d) and **p-biszigzag-HBC** (e and f).

To better understand the influence of zigzag edges on the HOMO–LUMO gaps and π-conjugation pathway, the nucleus-independent chemical shifts (NICS)^11^ and anisotropy of the induced current density (ACID)^12*a*^ were calculated to analyze the aromaticity of three isomers. As displayed in Fig. 3a, c and e, all three isomers displayed seven aromatic sextet rings showing largely negative NICS(1)_*zz*_ values (red color), which are consistent with the values of HBC. At the same time, they all possess two more aromatic rings (blue color) at the K-regions which contribute to the aromaticity of the whole system. ACID plots further emphasize the zigzag effect on the π-conjugated pathway. All three isomers possess 30 π-electrons and reveal diatropic ring currents (red color) for the outer periphery. **o-Biszigzag-HBC** displays seven localized benzene ring currents (Fig. 3b), which implies seven aromatic sextet rings and matches with the results of NICS calculations. In contrast, for **p-biszigzag-HBC**, there are two diatropic localized ring currents (Fig. 3f) around the two phenanthrene substructures (aromatic rings A–B–C and D–E–F) at the K-regions. For **m-biszigzag-HBC**, the NICS(1)_*zz*_ values of rings D and I are −3.8, which suggests a weak aromatic character. The broken paratropic ring currents (blue color) of rings D and I also imply a diminished aromatic character (Fig. 3d). A similar phenomenon was also observed for the ACID and NICS analysis of the central benzene ring of coronene. These results firmly demonstrate the pronounced effect of a zigzag edge on the HOMO–LUMO gaps and π-conjugation pathways.

### Optical properties

**Fig. 4:**
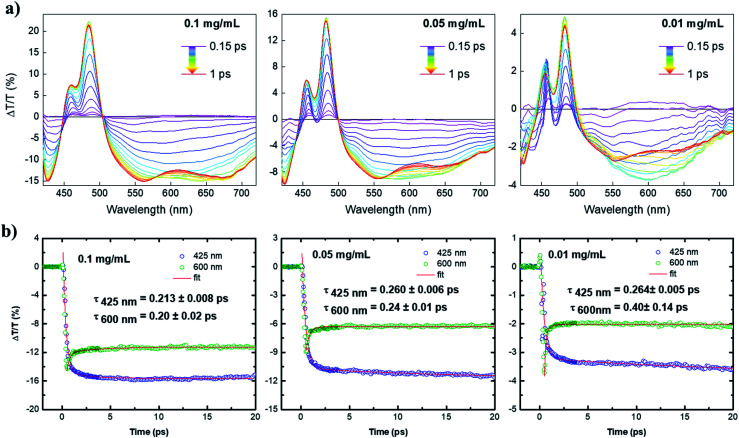
(a) TA spectra as a function of pump–probe delay (from 0.15 ps to 1 ps, taken every 0.05 ps) for **4** at three dilutions (0.10, 0.05, and 0.01 mg mL^−1^) in toluene solution; (b) exponential fitting of TA signal of the PA signal at a probe wavelength of 425 nm and 600 nm. Note that the peak at 600 nm is a convolution of a short-lived positive signal and a long-lived PA signal (the long-lived PA at 425 nm is reported for comparison).

With the help of ultrafast transient absorption (TA) spectroscopy, we could further obtain insights into the photophysical properties of **4**. The spectra of **4** in toluene were recorded at three concentrations, namely 1.2 × 10^−4^ mol L^−1^ (0.1 mg mL^−1^), 6 × 10^−5^ mol L^−1^ (0.05 mg mL^−1^), 1.2 × 10^−5^ mol L^−1^ (0.01 mg mL^−1^). The TA spectra as a function of pump–probe delay (from 150 fs to 1 ps) at the three dilutions are shown in Fig. 4a. The positive transient features lying at 480 and 450 nm could be assigned to depletion of the ground state due to the main HOMO → LUMO transition (photobleaching, PB) and its vibrionic replica, respectively. The negative signals lying at 425 nm and the lower energy region of the spectrum (from 500 nm) were ascribed to the photoinduced absorption (PA) from the first excited state S_1_ to higher excited states S_*i*_. By increasing the concentration, a progressive broadening of the positive transient peaks was observed, which is typical for aggregation of large PAHs.^13,14^ Interestingly, at the lowest dilution (0.01 mg mL^−1^), the large PA appears to incorporate a broad feature lying at 600 nm that overlaps with the calculated emission spectrum (see Fig. S1b†) and dominates the spectrum at early pump–probe delays. Such a signal can be related to stimulated emission (SE) that competes with the large and strong excited state absorption of **4**. The interplay between PA and SE is typical for large conjugated systems, in which SE is quenched due to the occurrence of PA transitions with charge-transfer (CT) character.^15^

Although in our case the molecule is rather small for supporting effective intramolecular charge separation, this can, in principle, occur *via* intermolecular charge separation due to effective π–π stacking of such planar molecules (*i.e.* supramolecular dimerization).^14^ By this scenario, the signal at 600 nm was greatly enhanced upon dilution to 0.01 mg mL^−1^, owing to the decrease of intermolecular CT-transitions in the most diluted solution. The overall decay kinetics at 600 nm (Fig. 4b) is a convolution of a short-lived positive signal that transforms into a very long-lived photoinduced absorption at 425 nm. The ultrafast component might be associated with an ultrafast phenomenon, *i.e.*, a π–π dimerization that would quench the SE at the expense of the PA signal with an intermolecular charge-transfer character. The observed two-fold increase of the PA at 600 nm upon dilution to 0.01 mg mL^−1^ corroborates the aforementioned scenario, as dilution can suppress the π–π dimerization and, in general, aggregation phenomena. Although the damping of SE is ultrafast, which is roughly 200 fs in the most diluted sample, these findings fully confirm the importance of zigzag edges to achieve optical gain properties in large PAHs with potential applications as organic laser materials.^13,14,16^ These findings have not been observed for fully armchair edged PAHs.

## Conclusions

In summary, we have achieved the synthesis of HBC **4** with two additional K-regions adopting an *ortho*-configuration. The vibrational and photophysical properties of **4** were studied by experimental and theoretical IR- and Raman spectra as well as by ultrafast transient absorption spectroscopy, providing insights into the edge structure engineering of the optoelectronic and photophysical properties of PAHs. This work offers a deeper understanding of the structure–property relationships of PAHs with extension in K-regions. The syntheses of further PAHs with unique edge structures are being pursued in our laboratory.

## Conflicts of interest

There are no conflicts to declare.

## Supplementary Material

SC-011-D0SC04649C-s001
