# Supplementary material for: Hexa-peri-benzocoronene with two extra K-regions in an ortho-configuration
Source: Chem Sci. 2020 Oct 23;11(47):12816–21. doi: 10.1039/d0sc04649c (PMC8163021; doi:10.1039/d0sc04649c)
Supplement: SC-011-D0SC04649C-s001 [file SC-011-D0SC04649C-s001.pdf]

Electronic Supplementary Material (ESI) for Chemical Science.
This journal is © The Royal Society of Chemistry 2020

Chemical Science

*Electronic Supporting Information*

# Hexa-*peri*-benzocoronene with two extra K-regions in an *ortho*-configuration

Tim Dumslaff,[‡,a] Yanwei Gu,[‡,a] Giuseppe M. Paternò,[‡,b] Zijie Qiu,[*,a] Ali Maghsoumi,[c] Matteo Tommasini,[c] Xinliang Feng,[d] Francesco Scotognella,[*,b] Akimitsu Narita,[a] Klaus Müllen[*,a]

[a.] Dr. T. Dumslaff, Dr. Y. Gu, Dr. Z. Qiu, Dr. A. Narita, Prof. Dr. K. Müllen, Max Planck Institute for Polymer Research, Ackermannweg 10, 55128, Mainz, Germany. E-mail: qiu@mpip-mainz.mpg.de, muellen@mpip-mainz.mpg.de

[b.] G. M. Paternò, Prof. F. Scotognella, Istituto Italiano di Tecnologia, Center for Nano Science and Technology, Milano, 20133, Italy. E-mail: francesco.scotognella@polimi.it

[c.] Ali Maghsoumi, Prof. M. Tommasini, Dipartimento di Chimica, Materiali e Ingegneria Chimica – Politecnico di Milano, Piazza Leonardo da Vinci, 32-20133 Milano, Italy

[d.] Prof. X. Feng, Center for Advancing Electronics Dresden (CFAED), Department of Chemistry and Food Chemistry, Dresden University of Technology, Walther-Hempel-Bau Mommsenstrasse 4, 01062 Dresden, Germany

‡ These authors contributed equally.

# Table of Contents

1. Experimental Section ........................................ S3

1.1 General ........................................ S3

1.2 Synthetic procedures and characterization data ........................................ S3

2. DFT Calculations ........................................ S5

3. Raman and IR Spectroscopies ........................................ S7

4. Ultrafast transient absorption (TA) spectroscopy ........................................ S9

5. $^{1}$H NMR, $^{13}$C NMR and mass spectra of all new compounds ........................................ S10

6. Cartesian coordinates of optimized structures for **4** and three biszigzag-HBCs. ........................................ S14

7. References ........................................ S21

# 1. Experimental Section

## 1.1 General

Unless otherwise noted, materials were purchased from Activate Scientific, Aldrich, Acros, ABCR, Merck, and Chempur, and used as received without further purification. All reactions dealing with air- or moisture-sensitive compounds have been carried out in a dry reaction vessel under argon protection. Preparative column chromatography has been performed on silica gel from Merck with a grain size of 0.063-0.200 mm (silica gel). UV-visible spectra were measured on a Perkin-Elmer Lambda 9 spectrophotometer at room temperature. Melting points were determined on a Büchi hot stage apparatus and were uncorrected. NMR spectra were measured on AVANCE 300 MHz Bruker spectrometer, and referenced to residual signals of the deuterated solvent. Abbreviations: s = singlet, d = doublet, dd = double doublet, t = triplet, m = multiplet. The high-resolution time-of-flight mass spectrometry were carried out on a rapifleXTM MALDI-TOF/TOF mass spectrometer from Bruker Daltonik GmbH, Fahrenheitstraße 4, 28359 Bremen. The instrument is equipped with a 10 kHz scanning smartbeam Nd:YAG laser at a wavelength of 355nm and a 10 bit 5 GHz digitizer.

## 1.2 Synthetic procedures and characterization data

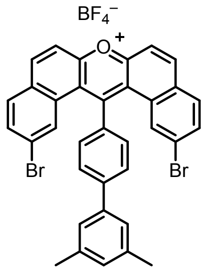


**1**

The synthesis of compound **1** was adopted from the method reported before.[1]

$^1$H NMR (500 MHz, tetrachloroethane-$d_2$): $\delta$ 8.73 (d, $J$ = 9.0 Hz, 2H), 8.24 (d, $J$ = 9.0 Hz, 2H), 8.12 (d, $J$ = 7.8 Hz, 2H), 7.99 (d, $J$ = 8.4 Hz, 2H), 7.86 (d, $J$ = 8.2 Hz, 2H), 7.52 (d, $J$ = 7.8 Hz, 2H), 7.41 (d, $J$ = 27.2 Hz, 4H), 7.12 (s, 1H), 2.42 (s, 6H).

$^{13}$C NMR (126 MHz, tetrachloroethane-$d_2$): $\delta$ 167.60, 159.45, 146.90, 146.37, 139.37, 138.92, 135.37, 133.07, 132.59, 131.45, 131.30, 130.93, 130.42, 129.74, 126.58, 126.18, 125.45, 121.13, 117.54, 21,47.

HRMS (MALDI-ToF, positive) *m/z* calcd for $C_{35}H_{23}Br_2O^+$ $[M]^+$ 617.0116, found 617.0127.

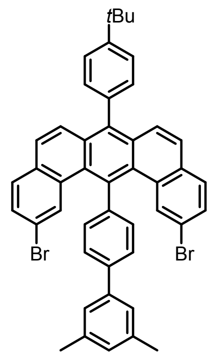


**2**

Compound **1** (2.00 g, 2.83 mmol) was suspended together with (*p*-*tert*-butylphenyl)acetic acid sodium salt (1.21 g, 5.66 mmol) in 20 g of benzoic anhydride, the reaction mixture was quickly colored from red to brown at 60 °C. The mixture was stirred under reflux at 160 °C for 24 h and then the solvent was distilled off under reduced pressure on a rotary evaporator (30 mbar, 82 °C). The brown residue was dissolved in dichloromethane and purified by column chromatography (DCM/PE = 1/4). The yellow product was then recrystallized from chloroform and precipitated with petroleum ether to give compound **2** in 56% yield.

$^{1}$H NMR (500 MHz, tetrachloroethane-$d_2$): $\delta$ 7.90 (d, $J$ = 8.0 Hz, 2H), 7.65 (d, $J$ = 8.3 Hz, 2H), 7.62 (d, $J$ = 8.2 Hz, 2H), 7.57 (s, 2H), 7.54 (d, $J$ = 8.2 Hz, 8H), 7.47 (s, 2H), 7.39 (d, $J$ = 8.1 Hz, 2H), 7.12 (s, 1H), 2.48 (s, 6H), 1.49 (s, 9H).

$^{13}$C NMR (126 MHz, tetrachloroethane-$d_2$): $\delta$ 150.63, 142.72, 142.24, 141.18, 138.32, 137.64, 137.51, 135.47, 132.59, 132.15, 132.02, 131.34, 131.23, 130.46, 129.70, 129.07, 128.97, 126.85, 126.77, 126.07, 125.61, 125.32, 118.46, 34.58, 31.41, 21.49.

HRMS (MALDI-ToF, positive) *m/z* calcd for $C_{46}H_{36}Br_2$ $[M]^+$ 746.1184, found 746.1182.

Melting point: 313-315 °C.

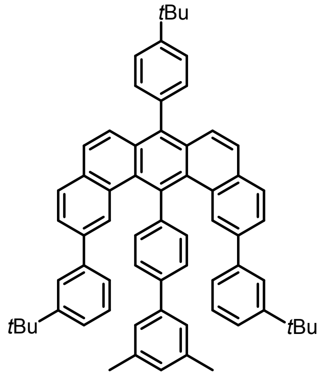


**3**

Compound **2** (0.16 g, 0.21 mmol) was placed together with 3-(*tert*-butyl)phenylboronic acid (0.228 g, 1.28 mmol) in a 100 mL round-bottomed flask and dried for one hour under high vacuum and then aerated with argon. 10 mL of toluene, 5 mL of ethanol and 1 mL of a 2M potassium carbonate solution were then added and the mixture was flushed with argon for one hour. $Pd(PPh_3)_4$ was added as a catalyst, the mixture was heated to 110 °C and stirred at this temperature for 24 h. It was cooled to room temperature and extracted with dichloromethane.

The organic phase was washed with saturated sodium chloride solution and dried over magnesium sulfate. The solvent was then distilled off under reduced pressure on a rotary evaporator. The yellow oily residue was dissolved in dichloromethane and purified by column chromatography (DCM/PE = 1/3). The product was recrystallized from dichloromethane to give compound **3** in 68% yield as a light-yellow solid.

$^1$H NMR (500 MHz, tetrachloroethane-$d_2$): $\delta$ 7.89 – 7.84 (m, 4H), 7.74 (d, J = 8.0 Hz, 2H), 7.71 – 7.67 (m, 6H), 7.61 (d, J = 9.1 Hz, 2H), 7.54 (d, J = 9.0 Hz, 2H), 7.49 (d, J = 8.1 Hz, 2H), 7.39 (s, 2H), 7.23 (d, J = 7.9 Hz, 2H), 7.10 (t, J = 7.7 Hz, 2H), 7.05 (d, J = 9.4 Hz, 3H), 6.97 (d, J = 7.6 Hz, 2H), 2.46 (s, 6H), 1.58 (s, 9H), 1.30 (s, 18H).

$^{13}$C NMR (126 MHz, tetrachloroethane-$d_2$): $\delta$ 151.07, 150.63, 143.92, 141.77, 140.78, 140.59, 137.54, 137.25, 137.18, 137.04, 136.20, 132.81, 132.20, 131.59, 131.21, 130.48, 129.21, 128.80, 128.68, 128.29, 128.07, 127.83, 126.64, 125.35, 125.18, 125.16, 125.03, 124.31, 123.99, 123.54, 34.50, 34.34, 31.35, 31.09, 21.07.

HRMS (MALDI-ToF, positive) *m/z* calcd for $C_{66}H_{62}$ $[M]^+$ 854.4852, found 842.4848.

Melting point: 353-355 °C.

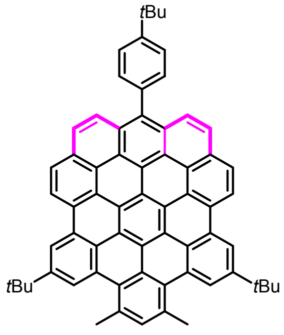


**4**

Compound **3** (15 mg, 14.65 μmol) and DDQ (30 mg, 132 μmol) was dissolved in stabilizer-free dichloromethane and stir at 0 ℃ for 10-15 mins. Then 0.1 mL trifluoromethane sulfonic acid was added in one portion. The reaction mixture was stirred under a constant flow of argon for 8 h and the reaction was terminated by adding methanol. The precipitate obtained was filtered off and washed with hydrochloric acid and methanol to provide **4** in 83% yield.

$^1$H NMR (700 MHz, THF-$d_8$/$CS_2$=2/1): $\delta$ 9.75 (d, J = 7.8 Hz, 2H), 9.55 (s, 2H), 9.18 (s, 2H), 8.80 (d, J = 7.9 Hz, 2H), 8.42 (d, J = 8.3 Hz, 2H), 8.36 (d, J = 8.6 Hz, 2H), 8.05 (s, 1H), 7.84 (d, J = 7.5 Hz, 2H), 7.59 (d, J = 7.1 Hz, 2H), 3.56 (s, 6H), 1.90 (s, 18H), 1.66 (s, 9H).

$^{13}$C NMR (176 MHz, THF-$d_8$/$CS_2$=2/1): $\delta$ 147.93, 137.81, 137.77, 134.63, 132.70, 131.68, 129.88, 129. 53, 128.56, 128.19, 127.81, 126.88, 126.37, 125.98, 124.48, 123.73, 123.44, 121.71, 121.08, 119.74, 119.27, 32.80, 32.37, 27.86.

HRMS (MALDI-ToF, positive) *m/z*. $C_{66}H_{50}[M]^+$ 842.3913, found 842.3948.

## 2. DFT Calculations

Theoretical calculations were performed with the Gaussian09 rev. D program suite.[2] All calculations were carried out using the density functional theory (DFT) method with Becke's

three-parameter hybrid exchange functionals and the Lee-Yang-Parr correlation functional (B3LYP) employing the 6-31G(d,p) basis set for all atoms.[3] NICS values were calculated using standard GIAO procedure.[4] ACID plot was calculated by using the method developed by Herges.[5] Theoretical Raman spectra at the B3LYP/6-31G** level work have been calculated and the wavenumber scaled down by a uniform factor of 0.96 such as commonly described for this level of theory.

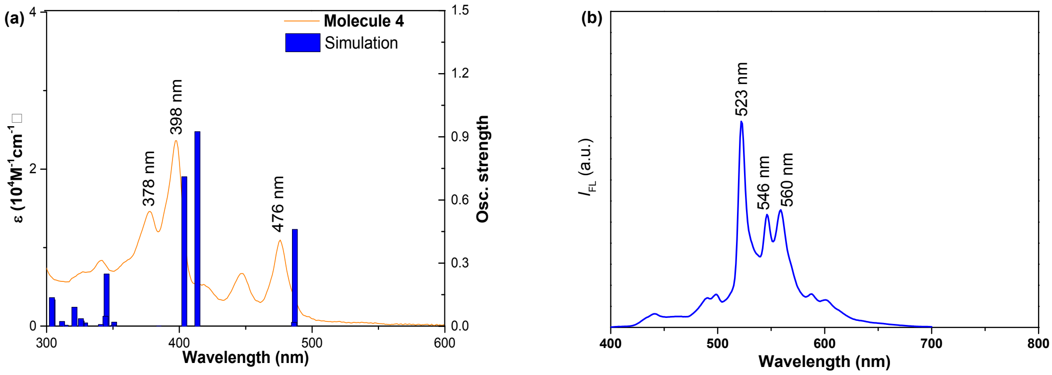


**Figure S1.** (a) Calculated absorption spectrum of **4** along with the experimental spectrum. (b) Calculated fluorescence emission of **4**.

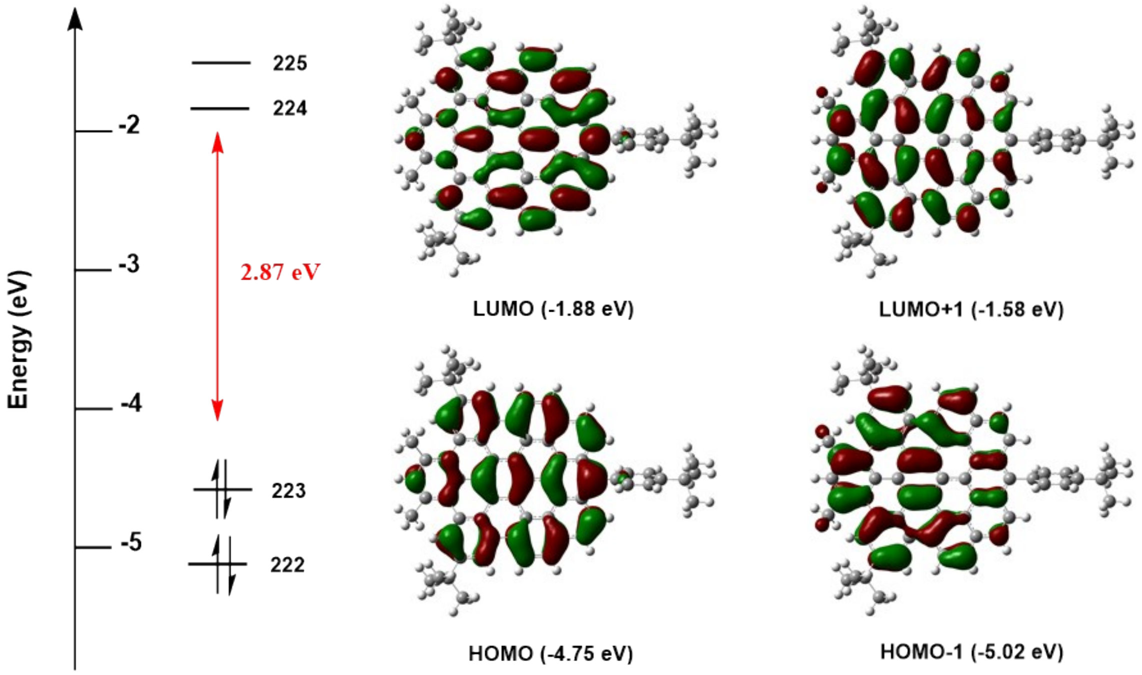


**Figure S2.** Frontier molecular orbital profiles and energy diagram of **4** obtained by B3LYP/6-31G(d,p) level calculations.

**Table S1.** Selected TDDFT (B3LYP/6-31G (d,p)) calculated wavelength, oscillator strength and compositions of major transitions of **4**.

| Wavelength (nm) | Osc. Strength | Major contributions | band |
|---|---|---|---|
| 487.5 | 0.46 | HOMO->LUMO (90%) | p |
| 487.0 | 0.0183 | H-1->LUMO (54%), HOMO->L+1 (41%) | α |
| 414.5 | 0.9247 | H-1->LUMO (42%), HOMO->L+1 (53%) | β |
| 404.7 | 0.3414 | H-1->L+1 (93%) | β' |

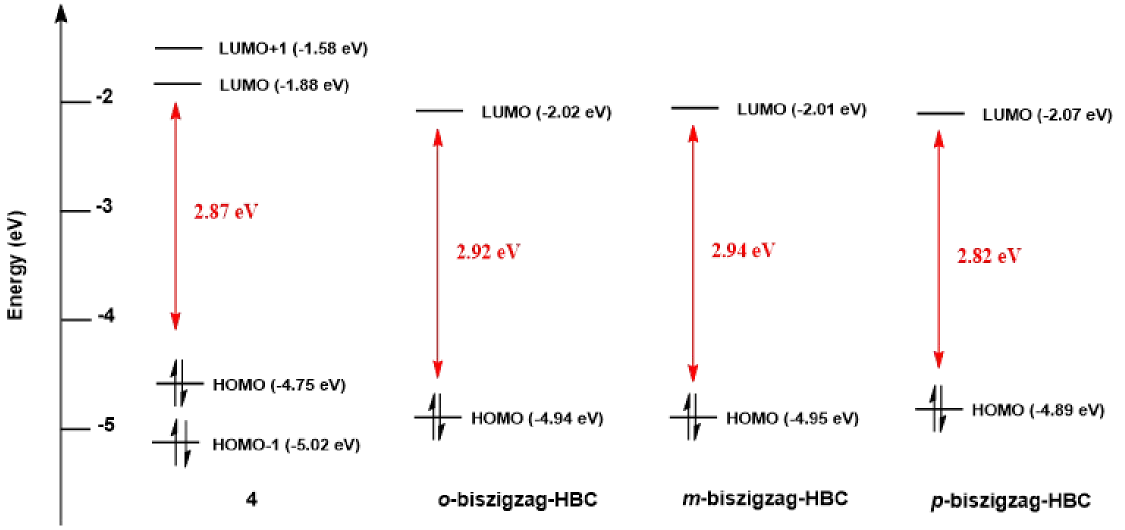


**Figure S3.** Frontier molecular orbital profiles of compound **4** and three biszigzag-HBC isomers by DFT calculations.

## 3. Raman and IR Spectroscopies

FT-Raman spectra have been recorded with a Nicolet NXR 9650 FT-Raman spectrometer collecting 4096 scans with a resolution of 4 $cm^{-1}$ on powder samples gently placed on a metallic sample holder. Laser excitation at 1064 nm has been kept focused on the sample (50 μm beam diameter) with a power of 50 mW. Micro FT-IR measurements were carried out with Nicolet Nexus equipment coupled with a Thermo-Nicolet Continuμm infrared microscope and a cooled MCT detector (77 K). Spectra were acquired on powder sampled by using the diamond anvil cell technique with a 15× infrared objective (256 scans, 4 $cm^{-1}$ resolution).

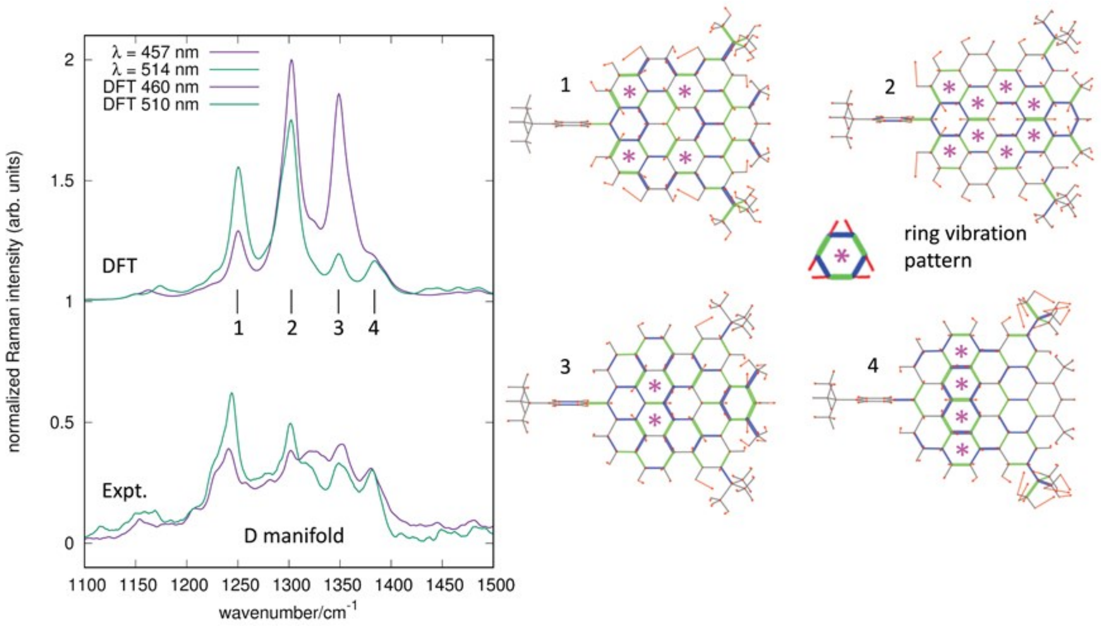


**Figure S4**: FT-Raman spectrum of **4** compared with results from DFT calculations over the D regions, which implies four ring vibration modes for skeleton.

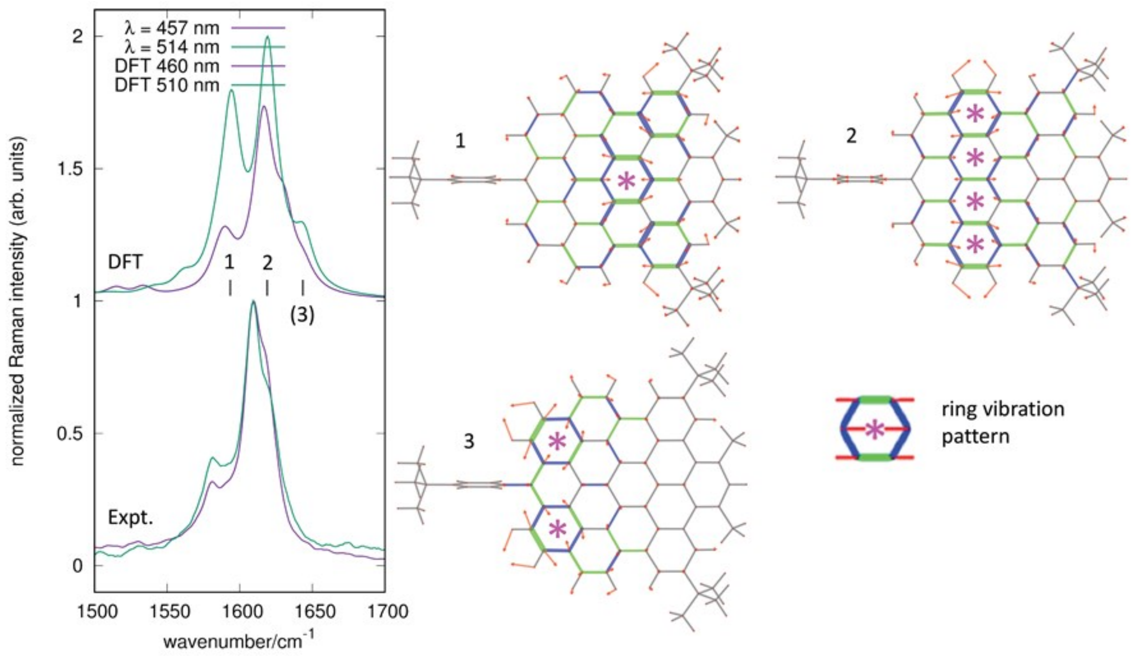


**Figure S5:** FT-Raman spectrum of **4** compared with results from DFT calculations over the G regions, which implies three ring vibration modes.

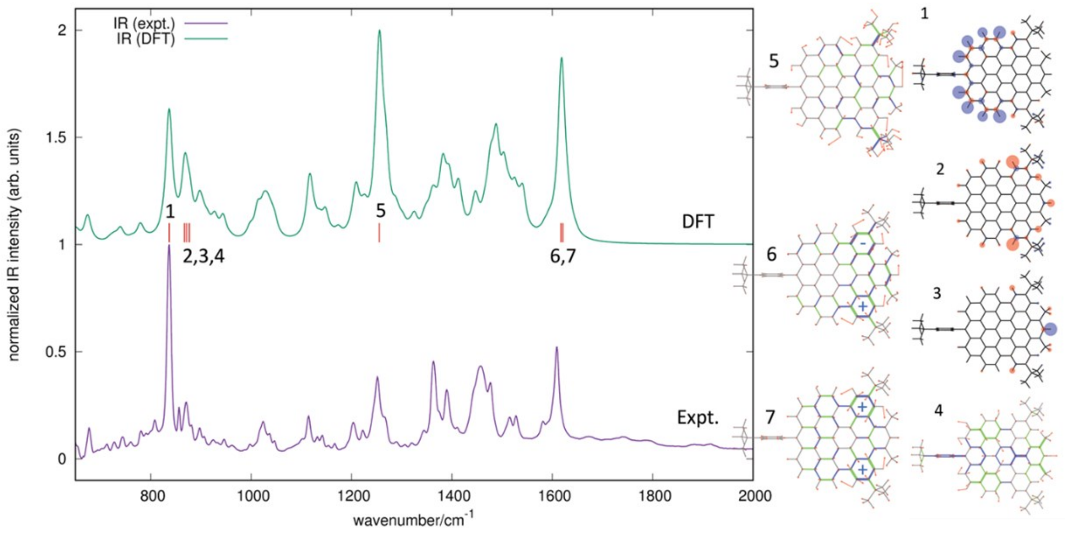


**Figure S6**: Micro IR spectrum of **4** and corresponding 7 vibration modes. Mode 5 shows the in-plane aromatic CH bending; Modes 6 and 7 are IR-active G modes showing out of phase and in phase G modes.

## 4. Ultrafast transient absorption (TA) spectroscopy

The pump and probe measurements were proceeded with an amplified Ti:sapphire laser with 2 mJ output energy, 2 kHz repetition rate and a central energy of 1.59 eV (800 nm). We used a pump wavelength of 400 nm excitation, which is resonant with the main π→π* transition. Such pump pulses were generated by frequency doubling of the amplified output of the Ti:sapphire laser using a barium borate crystal (BBO). Pump pulses were focused on a 200 µm spot (diameter), keeping pump fluences at ~ 2 mJ $cm^{-2}$. As a probe pulse, we used a broadband white light super-continuum generated in $CaF_2$ in the spectral region from 400 nm to 800 nm.

## 5. $^{1}$H NMR, $^{13}$C NMR and mass spectra of all new compounds

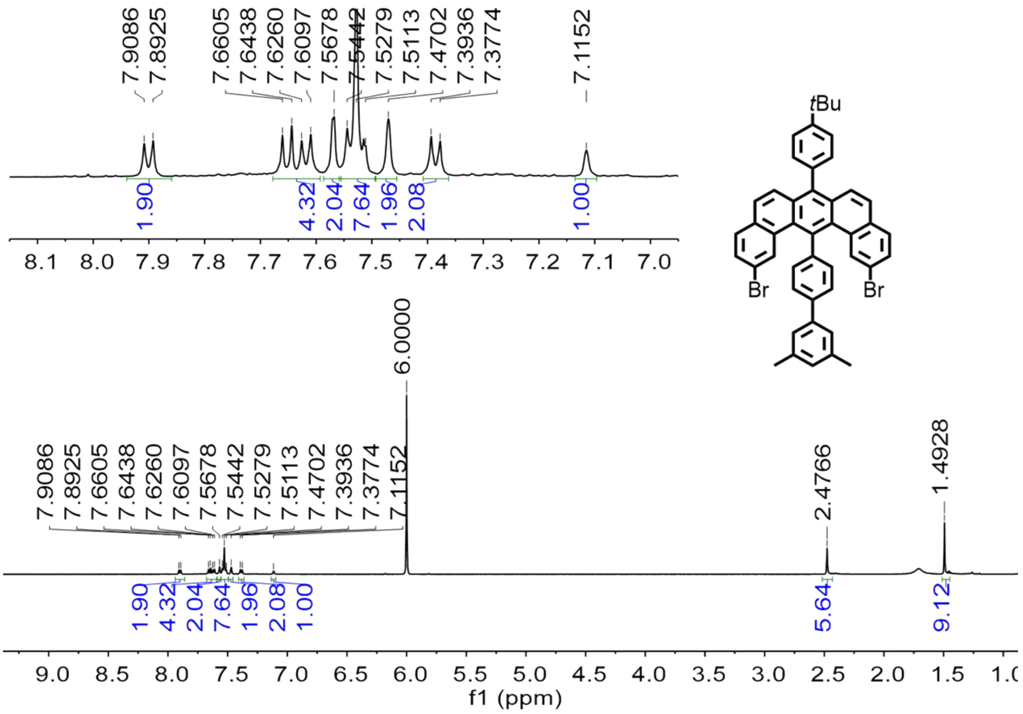


**Figure S7**. $^{1}$H NMR spectrum of compound **2** (500 MHz, tetrachloroethane-$d_2$).

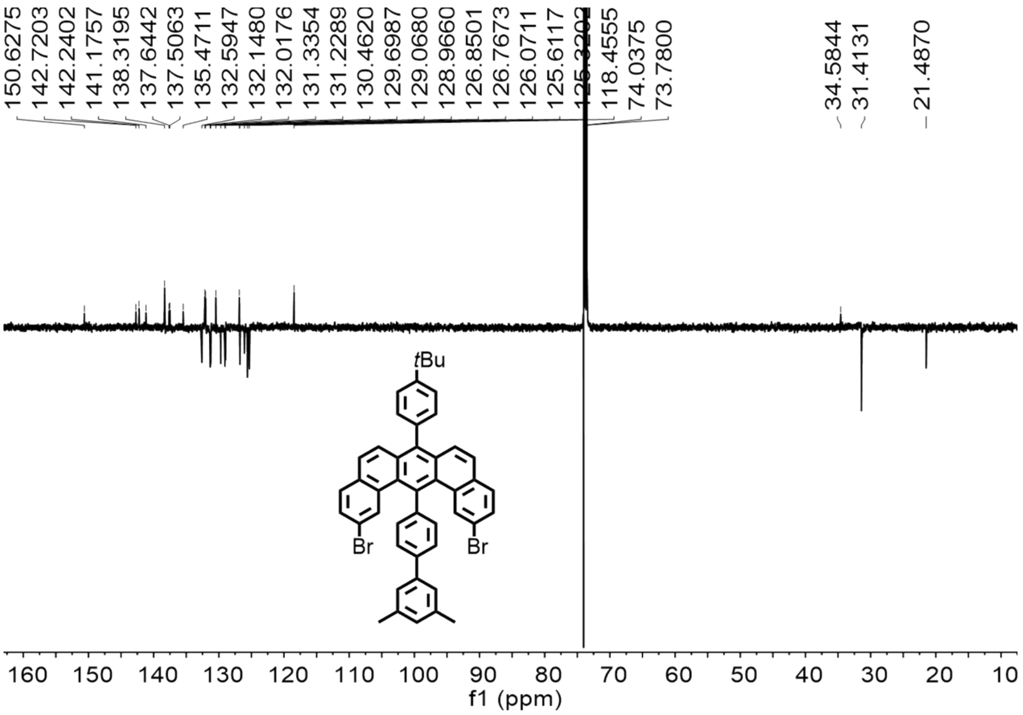


**Figure S8**. Spin-echo $^{13}$C NMR spectrum of compound **2** (126 MHz, tetrachloroethane-$d_2$).

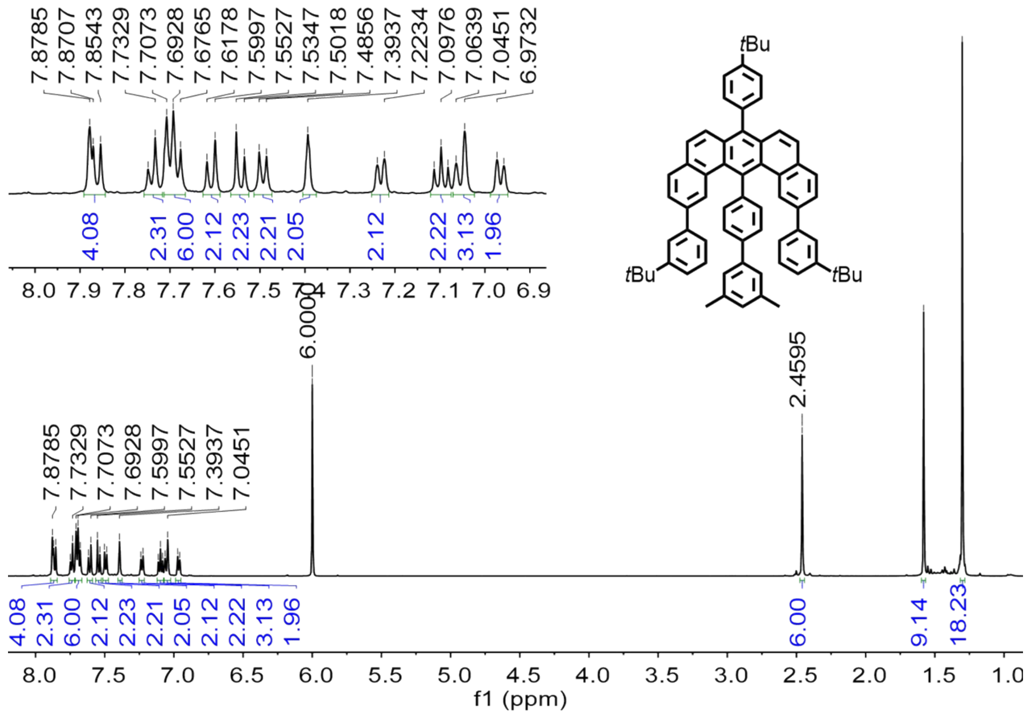

**Figure S9**. $^{1}$H NMR spectrum of compound **3** (500 MHz, tetrachloroethane-$d_2$).

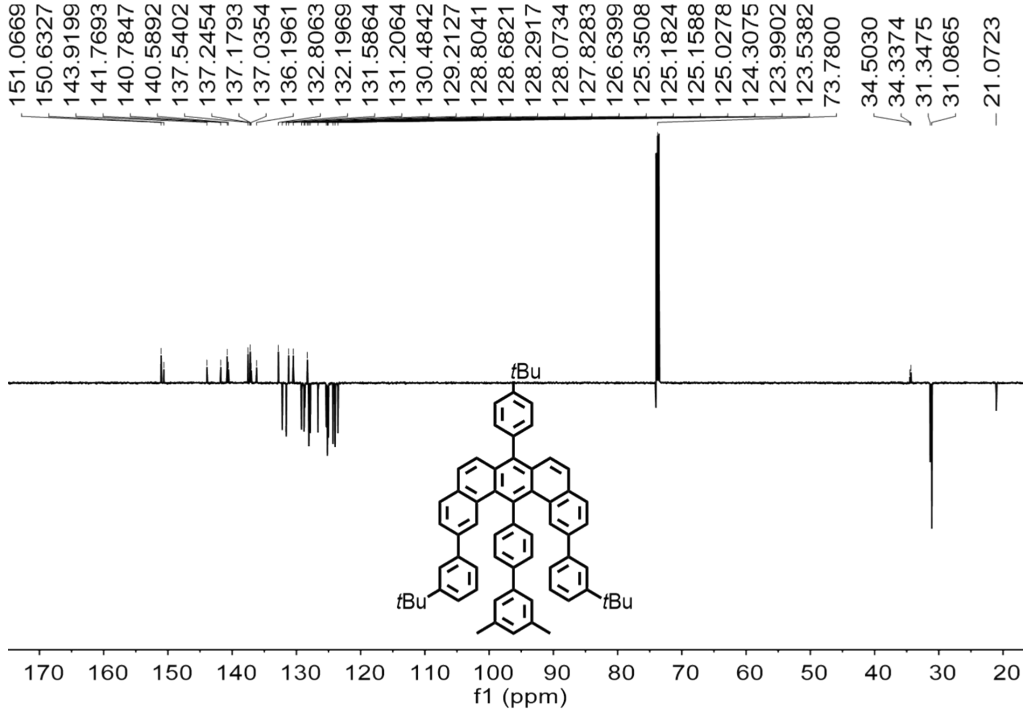

**Figure S10**. Spin-echo $^{13}$C NMR spectrum of compound **3** (126 MHz, tetrachloroethane-$d_2$).

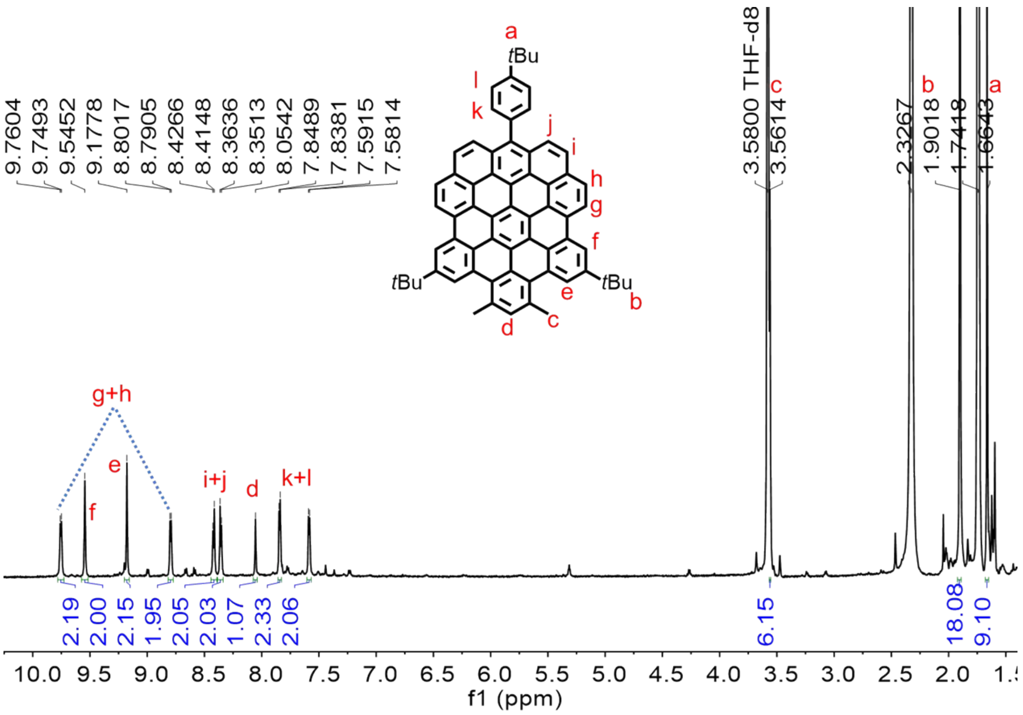


**Figure S11**. $^{1}$H NMR spectrum of compound **4** (700 MHz, tetrahydrofuran-$d_8$/$CS_2$=2/1).

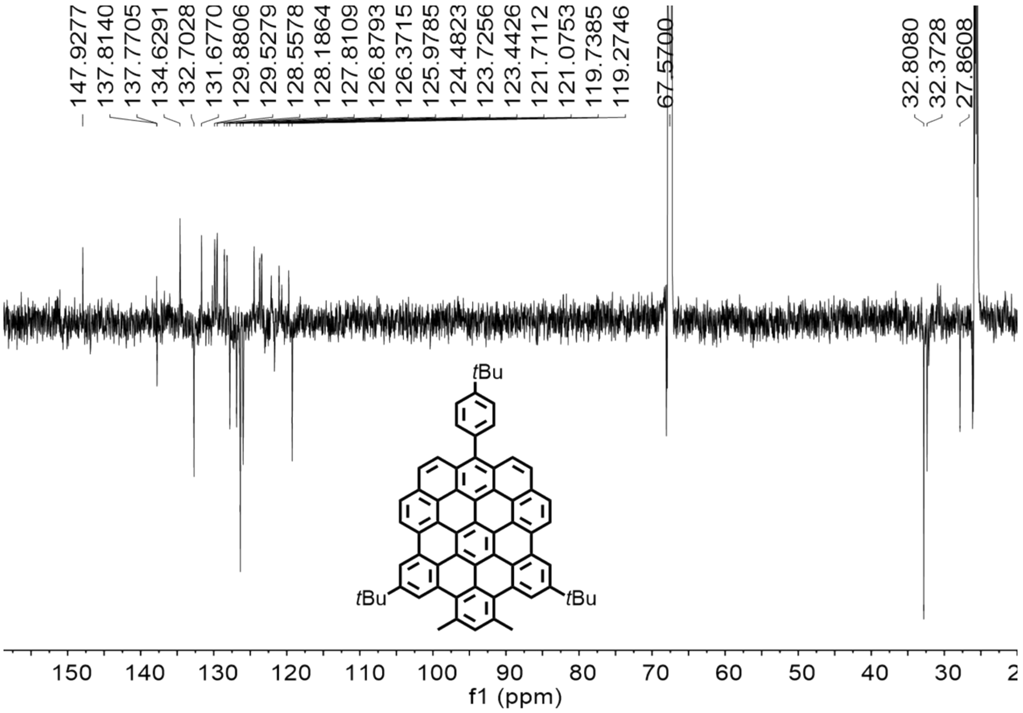


**Figure S12**. Spin-echo $^{13}$C NMR spectrum of compound **4** (176 MHz, tetrahydrofuran-$d_8$/$CS_2$=2/1).

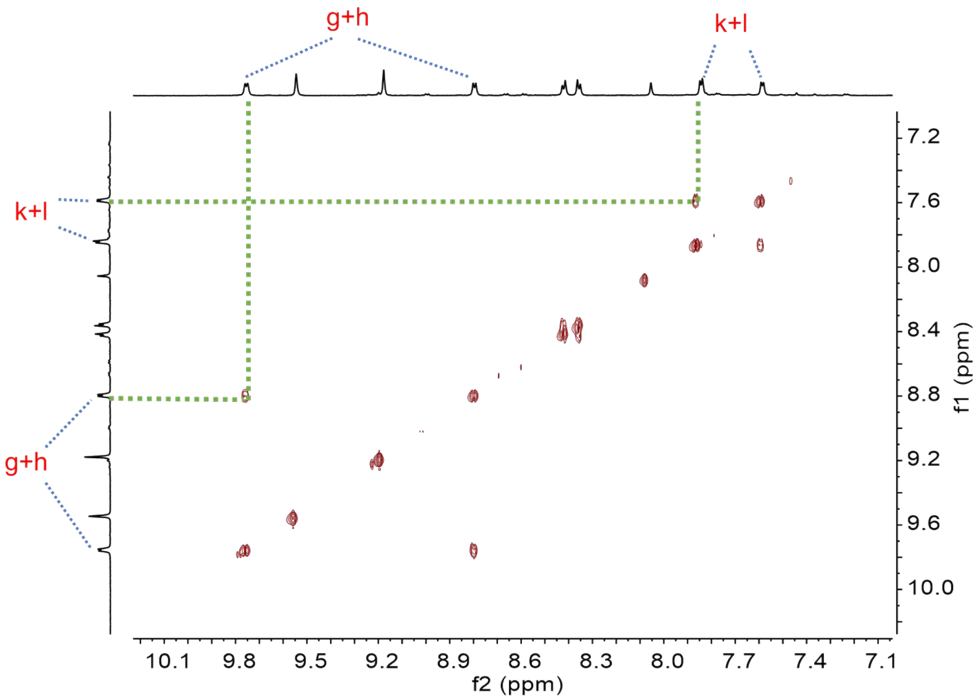


**Figure S13**. Aromatic region of the $^{1}$H-$^{1}$H COSY spectrum of **4** measured at room temperature (700 MHz, tetrahydrofuran-$d_8$/$CS_2$=2/1).

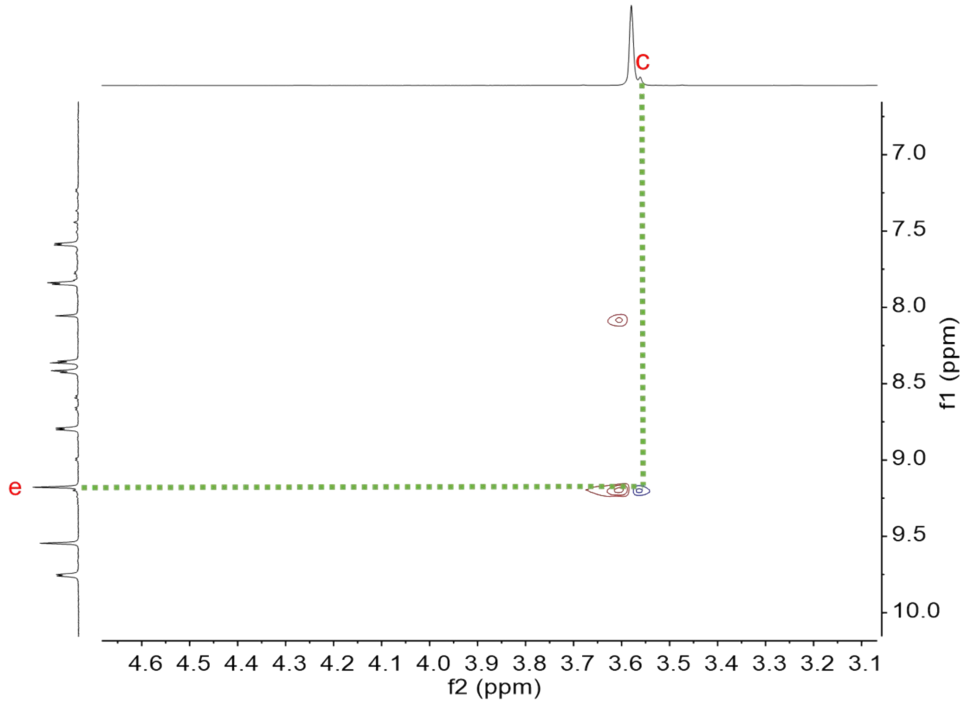


**Figure S14**. Aliphatic and aromatic region of the $^{1}$H-$^{1}$H NOESY spectrum of **4** measured at room temperature (700 MHz, tetrahydrofuran-$d_8$/$CS_2$=2/1).

6. **Cartesian coordinates of optimized structures for 4 and three biszigzag-HBCs.**

**Table S2.** Cartesian coordinates of optimized structure for **4**.

| | X | Y | Z |
|---|---|---|---|
| C | 3.799076 | -1.215146 | -0.207670 |
| C | 2.365104 | -1.216254 | -0.191024 |
| C | 1.652256 | -0.005352 | -0.010183 |
| C | 2.369352 | 1.203406 | 0.168157 |
| C | 3.803438 | 1.198025 | 0.180899 |
| C | 4.506927 | -0.009498 | -0.014222 |
| C | 1.656415 | 2.432221 | 0.328745 |
| C | 2.376383 | 3.642582 | 0.524979 |
| C | 3.805331 | 3.606221 | 0.567876 |
| C | 4.488949 | 2.441724 | 0.399550 |
| C | 4.480299 | -2.460465 | -0.428679 |
| C | 3.792529 | -3.622822 | -0.596491 |
| C | 2.363824 | -3.654709 | -0.550435 |
| C | 1.647938 | -2.442873 | -0.350808 |
| C | 0.228592 | 2.450420 | 0.276158 |
| C | -0.459577 | 3.684866 | 0.363659 |
| C | 0.285382 | 4.864709 | 0.588669 |
| C | 1.659820 | 4.845678 | 0.674324 |
| C | 1.642987 | -4.855842 | -0.699693 |
| C | 0.269232 | -4.871426 | -0.610952 |
| C | -0.472879 | -3.689372 | -0.382468 |
| C | 0.219984 | -2.457103 | -0.293944 |
| C | 0.211844 | -0.003287 | -0.007239 |
| C | -0.502659 | -1.222449 | -0.141506 |
| C | -1.926229 | -1.221766 | -0.124151 |
| C | -2.645321 | -0.000095 | 0.002891 |
| C | -1.922645 | 1.219248 | 0.122468 |
| C | -0.498411 | 1.216913 | 0.129666 |
| C | -1.919309 | -3.704158 | -0.216651 |
| C | -1.907469 | 3.703797 | 0.201156 |
| C | -4.097876 | 0.002192 | 0.010191 |
| C | -4.807189 | -1.238895 | -0.100858 |
| C | -6.215893 | -1.192410 | -0.256839 |
| C | -6.860850 | 0.007751 | 0.027094 |
| C | -6.208736 | 1.205325 | 0.301173 |
| C | -4.801230 | 1.246174 | 0.127059 |

| | | | |
|---|---|---|---|
| C | 6.004325 | -0.011844 | -0.014989 |
| C | 6.729281 | 0.321584 | -1.164369 |
| C | 8.126161 | 0.319185 | -1.163110 |
| C | 8.857331 | -0.014195 | -0.015404 |
| C | 8.121019 | -0.347042 | 1.134455 |
| C | 6.728721 | -0.347996 | 1.138481 |
| C | -7.106758 | -2.317111 | -0.749218 |
| C | -7.087900 | 2.334557 | 0.804550 |
| C | -2.618136 | -4.914368 | -0.063603 |
| C | -3.986351 | -4.950044 | 0.176770 |
| C | -4.675091 | -3.730119 | 0.179732 |
| C | -4.052538 | -2.495996 | -0.050138 |
| C | -2.631777 | -2.473496 | -0.160588 |
| C | -2.625399 | 2.472792 | 0.158670 |
| C | -4.043812 | 2.499507 | 0.062199 |
| C | -4.670874 | 3.735905 | -0.173235 |
| C | -3.978722 | 4.948194 | -0.191438 |
| C | -2.604229 | 4.908284 | 0.040251 |
| C | 10.396352 | -0.025816 | 0.025080 |
| C | 11.019444 | 0.362328 | -1.328922 |
| C | 10.892224 | -1.444099 | 0.394022 |
| C | 10.891242 | 0.981634 | 1.089977 |
| C | -4.752976 | -6.257180 | 0.454635 |
| C | -3.848204 | -7.500187 | 0.360471 |
| C | -5.896044 | -6.419969 | -0.575128 |
| C | -5.351659 | -6.204482 | 1.880608 |
| C | -4.669272 | 6.298110 | -0.465585 |
| C | -6.182341 | 6.147910 | -0.712967 |
| C | -4.042148 | 6.952999 | -1.719238 |
| C | -4.472778 | 7.231380 | 0.752787 |
| H | 4.340655 | 4.537647 | 0.732473 |
| H | 5.572073 | 2.436841 | 0.427644 |
| H | 5.563372 | -2.458831 | -0.459780 |
| H | 4.324606 | -4.555726 | -0.763257 |
| H | -0.227524 | 5.810147 | 0.716806 |
| H | 2.207198 | 5.767394 | 0.852353 |
| H | 2.187451 | -5.778866 | -0.879943 |
| H | -0.245688 | -5.815692 | -0.738710 |
| H | -7.949214 | 0.009111 | 0.035249 |
| H | 6.194784 | 0.584763 | -2.072814 |
| H | 8.639815 | 0.582877 | -2.080517 |
| H | 8.640689 | -0.611437 | 2.050754 |

| | | | |
|---|---|---|---|
| H | 6.192337 | -0.609834 | 2.046162 |
| H | -6.587904 | -2.982725 | -1.442039 |
| H | -7.518462 | -2.933955 | 0.059411 |
| H | -7.962147 | -1.884618 | -1.276682 |
| H | -7.935533 | 1.906113 | 1.347691 |
| H | -6.555425 | 3.000586 | 1.486599 |
| H | -7.510650 | 2.950541 | 0.001376 |
| H | -2.062164 | -5.840335 | -0.073604 |
| H | -5.726020 | -3.747876 | 0.413518 |
| H | -5.722312 | 3.746629 | -0.395710 |
| H | -2.049080 | 5.837584 | 0.033348 |
| H | 10.732656 | 1.373474 | -1.635726 |
| H | 12.111263 | 0.338963 | -1.252609 |
| H | 10.729937 | -0.331818 | -2.124550 |
| H | 10.516470 | -1.766962 | 1.369400 |
| H | 11.986996 | -1.465682 | 0.435454 |
| H | 10.566168 | -2.178885 | -0.349294 |
| H | 11.986225 | 0.982127 | 1.134009 |
| H | 10.560434 | 1.997732 | 0.851432 |
| H | 10.518551 | 0.735381 | 2.088534 |
| H | -3.040950 | -7.475079 | 1.099516 |
| H | -4.440074 | -8.401034 | 0.551471 |
| H | -3.401262 | -7.604167 | -0.633767 |
| H | -6.612407 | -5.594742 | -0.526841 |
| H | -6.447936 | -7.347863 | -0.387526 |
| H | -5.501513 | -6.460590 | -1.595727 |
| H | -5.912055 | -7.121970 | 2.093011 |
| H | -4.561838 | -6.106395 | 2.632283 |
| H | -6.035910 | -5.359995 | 2.005096 |
| H | -6.696964 | 5.712873 | 0.150024 |
| H | -6.623685 | 7.132665 | -0.896896 |
| H | -6.392890 | 5.525065 | -1.588601 |
| H | -2.971131 | 7.134764 | -1.592210 |
| H | -4.521118 | 7.916633 | -1.926449 |
| H | -4.169260 | 6.313986 | -2.599021 |
| H | -4.954898 | 8.198922 | 0.573353 |
| H | -4.911955 | 6.795054 | 1.655802 |
| H | -3.414590 | 7.420660 | 0.955572 |

**Table S3.** Cartesian coordinates of optimized structure for ***o*-biszigzag-HBC**

| | X | Y | Z |
|---|---|---|---|
| C | 4.540250 | 1.220167 | -0.000001 |
| C | 3.107321 | 1.224795 | 0.000008 |
| C | 2.393799 | 0.000001 | -0.000007 |
| C | 3.107321 | -1.224792 | -0.000046 |
| C | 4.540249 | -1.220164 | -0.000069 |
| C | 5.221841 | 0.000002 | -0.000046 |
| C | 2.399863 | 2.464137 | 0.000044 |
| C | 0.971206 | 2.476573 | 0.000032 |
| C | 0.245554 | 1.232369 | 0.000018 |
| C | 0.954476 | 0.000001 | -0.000006 |
| C | 0.245554 | -1.232368 | 0.000005 |
| C | 0.971208 | -2.476572 | -0.000016 |
| C | 2.399865 | -2.464136 | -0.000050 |
| C | 3.130068 | 3.685648 | 0.000076 |
| C | 2.416038 | 4.899043 | 0.000110 |
| C | 1.039043 | 4.910155 | 0.000096 |
| C | 0.282496 | 3.715191 | 0.000053 |
| C | -1.177501 | 1.233470 | 0.000001 |
| C | -1.888391 | 0.000000 | 0.000005 |
| C | -1.177499 | -1.233472 | 0.000008 |
| C | 0.282498 | -3.715190 | 0.000007 |
| C | 1.039048 | -4.910155 | -0.000043 |
| C | 2.416042 | -4.899042 | -0.000091 |
| C | 3.130071 | -3.685644 | -0.000089 |
| C | -1.890659 | 2.487499 | -0.000003 |
| C | -3.316639 | 2.499096 | -0.000044 |
| C | -4.049859 | 1.232612 | -0.000078 |
| C | -3.332940 | -0.000002 | -0.000027 |
| C | -4.049857 | -1.232615 | -0.000023 |
| C | -3.316636 | -2.499100 | 0.000073 |
| C | -1.890656 | -2.487500 | 0.000046 |
| C | -5.452246 | 1.202189 | -0.000162 |
| C | -6.144471 | -0.000003 | -0.000192 |
| C | -5.452246 | -1.202193 | -0.000124 |
| C | -1.174677 | 3.724261 | 0.000020 |
| C | -1.898690 | 4.927863 | 0.000008 |
| C | -3.284686 | 4.930309 | -0.000024 |
| C | -3.985386 | 3.731268 | -0.000050 |
| C | -3.985381 | -3.731272 | 0.000193 |
| C | -3.284679 | -4.930312 | 0.000256 |

| | | | |
|---|---|---|---|
| C | -1.898684 | -4.927865 | 0.000190 |
| C | -1.174672 | -3.724263 | 0.000079 |
| C | 4.561708 | 3.652701 | 0.000074 |
| C | 5.238849 | 2.472046 | 0.000033 |
| C | 5.238850 | -2.472040 | -0.000114 |
| C | 4.561710 | -3.652697 | -0.000126 |
| H | 6.309157 | 0.000002 | -0.000061 |
| H | 2.966493 | 5.836017 | 0.000144 |
| H | 0.536013 | 5.868970 | 0.000120 |
| H | 0.536017 | -5.868970 | -0.000055 |
| H | 2.966499 | -5.836015 | -0.000132 |
| H | -6.020168 | 2.123618 | -0.000219 |
| H | -7.230337 | -0.000003 | -0.000274 |
| H | -6.020165 | -2.123623 | -0.000167 |
| H | -1.379449 | 5.877711 | 0.000023 |
| H | -3.824957 | 5.872264 | -0.000030 |
| H | -5.067130 | 3.767529 | -0.000071 |
| H | -5.067125 | -3.767535 | 0.000262 |
| H | -3.824949 | -5.872268 | 0.000360 |
| H | -1.379442 | -5.877713 | 0.000244 |
| H | 5.099002 | 4.597338 | 0.000107 |
| H | 6.325342 | 2.456862 | 0.000027 |
| H | 6.325344 | -2.456856 | -0.000140 |
| H | 5.099007 | -4.597334 | -0.000160 |

**Table S4.** Cartesian coordinates of optimized structure for ***m*-biszigzag-HBC**.

| | X | Y | Z |
|---|---|---|---|
| C | -3.572189 | -3.436911 | 0.000064 |
| C | -2.852054 | -2.209732 | 0.000036 |
| C | -1.423125 | -2.213171 | 0.000011 |
| C | -0.724100 | -3.447915 | -0.000004 |
| C | -1.475018 | -4.653437 | 0.000017 |
| C | -2.846547 | -4.650605 | 0.000053 |
| C | -3.568277 | -0.974219 | 0.000040 |
| C | -2.863685 | 0.268969 | 0.000005 |
| C | -1.422007 | 0.271007 | 0.000004 |
| C | -0.710367 | -0.963528 | -0.000004 |
| C | 0.710366 | -0.963528 | -0.000008 |
| C | 1.423125 | -2.213170 | -0.000029 |
| C | 0.724102 | -3.447914 | -0.000036 |
| C | -4.991826 | -0.991612 | 0.000068 |
| C | -5.685080 | 0.239172 | 0.000067 |

| | | | |
|---|---|---|---|
| C | -5.005250 | 1.431606 | 0.000023 |
| C | -3.587824 | 1.484602 | -0.000014 |
| C | -0.713683 | 1.502088 | -0.000005 |
| C | 0.713682 | 1.502089 | 0.000008 |
| C | 1.422007 | 0.271006 | 0.000001 |
| C | 2.852055 | -2.209731 | -0.000044 |
| C | 3.572191 | -3.436909 | -0.000076 |
| C | 2.846549 | -4.650605 | -0.000092 |
| C | 1.475021 | -4.653437 | -0.000070 |
| C | -1.438523 | 2.747739 | -0.000038 |
| C | -0.732282 | 3.987939 | -0.000062 |
| C | 0.732281 | 3.987938 | 0.000038 |
| C | 1.438521 | 2.747739 | 0.000034 |
| C | 2.867430 | 2.748804 | 0.000079 |
| C | 3.587822 | 1.484604 | 0.000049 |
| C | 2.863685 | 0.268971 | 0.000008 |
| C | 1.462764 | 5.183173 | 0.000144 |
| C | 2.852664 | 5.178884 | 0.000202 |
| C | 3.546080 | 3.980011 | 0.000157 |
| C | -2.867430 | 2.748803 | -0.000074 |
| C | -3.546081 | 3.980011 | -0.000181 |
| C | -2.852667 | 5.178883 | -0.000249 |
| C | -1.462765 | 5.183173 | -0.000188 |
| C | 5.005250 | 1.431607 | 0.000051 |
| C | 5.685081 | 0.239175 | 0.000014 |
| C | 4.991825 | -0.991611 | -0.000022 |
| C | 3.568277 | -0.974218 | -0.000021 |
| C | -5.683372 | -2.238770 | 0.000097 |
| C | -4.997906 | -3.419240 | 0.000096 |
| C | 4.997907 | -3.419238 | -0.000087 |
| C | 5.683373 | -2.238766 | -0.000060 |
| H | -0.962750 | -5.607366 | 0.000009 |
| H | -3.393323 | -5.589714 | 0.000073 |
| H | -6.771696 | 0.231413 | 0.000100 |
| H | -5.580141 | 2.349019 | 0.000030 |
| H | 3.393325 | -5.589714 | -0.000119 |
| H | 0.962750 | -5.607366 | -0.000080 |
| H | 0.951852 | 6.137495 | 0.000201 |
| H | 3.395689 | 6.119263 | 0.000282 |
| H | 4.628118 | 4.007478 | 0.000198 |
| H | -4.628118 | 4.007476 | -0.000228 |
| H | -3.395690 | 6.119263 | -0.000352 |

| | | | |
|---|---|---|---|
| H | -0.951854 | 6.137494 | -0.000263 |
| H | 5.580137 | 2.349022 | 0.000079 |
| H | 6.771696 | 0.231416 | 0.000015 |
| H | -6.769978 | -2.229299 | 0.000122 |
| H | -5.528688 | -4.367474 | 0.000119 |
| H | 5.528691 | -4.367471 | -0.000117 |
| H | 6.769978 | -2.229296 | -0.000067 |

**Table S5.** Cartesian coordinates of optimized structure for ***p*-biszigzag-HBC**.

| | X | Y | Z |
|---|---|---|---|
| C | -1.203403 | -4.987419 | 0.000443 |
| C | -1.235302 | -3.583872 | 0.000185 |
| C | 0.000000 | -2.864747 | 0.000123 |
| C | 1.235303 | -3.583873 | 0.000202 |
| C | 1.203403 | -4.987418 | 0.000464 |
| C | -0.000001 | -5.678215 | 0.000597 |
| C | -2.493551 | -2.849760 | 0.000000 |
| C | -2.478296 | -1.434597 | -0.000036 |
| C | -1.231167 | -0.710746 | 0.000009 |
| C | 0.000000 | -1.425227 | 0.000035 |
| C | 1.231167 | -0.710746 | 0.000017 |
| C | 2.478297 | -1.434596 | -0.000022 |
| C | 2.493553 | -2.849759 | 0.000027 |
| C | -3.745055 | -3.515171 | -0.000151 |
| C | -4.932650 | -2.825336 | -0.000272 |
| C | -4.951370 | -1.413872 | -0.000251 |
| C | -3.711605 | -0.714051 | -0.000142 |
| C | -1.231168 | 0.710746 | 0.000020 |
| C | 0.000000 | 1.425227 | 0.000042 |
| C | 1.231167 | 0.710746 | 0.000020 |
| C | 3.711604 | -0.714051 | -0.000127 |
| C | 4.951371 | -1.413873 | -0.000231 |
| C | 4.932651 | -2.825334 | -0.000237 |
| C | 3.745055 | -3.515171 | -0.000112 |
| C | -2.478295 | 1.434597 | -0.000015 |
| C | -2.493551 | 2.849759 | 0.000047 |
| C | -1.235304 | 3.583873 | 0.000185 |
| C | 0.000000 | 2.864746 | 0.000126 |
| C | 1.235303 | 3.583872 | 0.000185 |
| C | 2.493550 | 2.849760 | 0.000033 |
| C | 2.478296 | 1.434596 | -0.000016 |
| C | -1.203404 | 4.987418 | 0.000389 |

| | | | |
|---|---|---|---|
| C | 0.000000 | 5.678215 | 0.000507 |
| C | 1.203403 | 4.987419 | 0.000397 |
| C | -3.711605 | 0.714051 | -0.000120 |
| C | -4.951370 | 1.413871 | -0.000198 |
| C | -4.932650 | 2.825336 | -0.000163 |
| C | -3.745056 | 3.515171 | -0.000042 |
| C | 3.745054 | 3.515172 | -0.000085 |
| C | 4.932650 | 2.825336 | -0.000208 |
| C | 4.951370 | 1.413872 | -0.000219 |
| C | 3.711605 | 0.714051 | -0.000124 |
| C | -6.176482 | -0.682051 | -0.000343 |
| C | -6.176482 | 0.682051 | -0.000315 |
| C | 6.176482 | -0.682051 | -0.000337 |
| C | 6.176482 | 0.682052 | -0.000331 |
| H | -2.124939 | -5.555388 | 0.000568 |
| H | 2.124939 | -5.555388 | 0.000609 |
| H | 0.000001 | -6.764141 | 0.000830 |
| H | -3.781106 | -4.597241 | -0.000199 |
| H | -5.875840 | -3.364973 | -0.000394 |
| H | 5.875840 | -3.364973 | -0.000349 |
| H | 3.781107 | -4.597241 | -0.000150 |
| H | -2.124939 | 5.555387 | 0.000482 |
| H | -0.000001 | 6.764140 | 0.000688 |
| H | 2.124938 | 5.555387 | 0.000509 |
| H | -5.875841 | 3.364972 | -0.000235 |
| H | -3.781106 | 4.597242 | -0.000036 |
| H | 3.781106 | 4.597241 | -0.000105 |
| H | 5.875840 | 3.364973 | -0.000304 |
| H | -7.111255 | -1.236173 | -0.000434 |
| H | -7.111255 | 1.236172 | -0.000381 |
| H | 7.111257 | -1.236170 | -0.000421 |
| H | 7.111255 | 1.236172 | -0.000410 |

## 7. References

[1] P. Ruffieux, S. Wang, B. Yang, C. Sánchez-Sánchez, J. Liu, T. Dienel, L. Talirz, P. Shinde, C. A. Pignedoli, D. Passerone, T. Dumslaff, X. Feng, K. Müllen, R. Fasel, Nature, 2016, **531**, 489-492.

[2] a) Gaussian 09; Revision A.2; Frisch, M. J.; Trucks, G. W.; Schlegel, H. B.; Scuseria, G. E.; Robb, M. A.; Cheeseman, J. R.; Scalmani, G.; Barone, V.; Mennucci, B.; Petersson, G. A.;

Nakatsuji, H.; Caricato, M.; Li, X.; Hratchian, H. P.; Izmaylov, A. F.; Bloino, J.; Zheng, G.; Sonnenberg, J. L.; Hada, M.; Ehara, M.; Toyota, K.; Fukuda, R.; Hasegawa, J.; Ishida, M.; Nakajima, T.; Honda, Y.; Kitao, O.; Nakai, H.; Vreven, T.; Montgomery, J., J. A.; Peralta, J. E.; Ogliaro, F.; Bearpark, M.; Heyd, J. J.; Brothers, E.; Kudin, K. N.; Staroverov, V. N.; Kobayashi, R.; Normand, J.; Raghavachari, K.; Rendell, A.; Burant, J. C.; Iyengar, S. S.; Tomasi, J.; Cossi, M.; Rega, N.; Millam, N. J.; Klene, M.; Knox, J. E.; Cross, J. B.; Bakken, V.; Adamo, C.; Jaramillo, J.; Gomperts, R.; Stratmann, R. E.; Yazyev, O.; Austin, A. J.; Cammi, R.; Pomelli, C.; Ochterski, J. W.; Martin, R. L.; Morokuma, K.; Zakrzewski, V. G.; Voth, G. A.; Salvador, P.; Dannenberg, J. J.; Dapprich, S.; Daniels, A. D.; Farkas, Ö.; Foresman, J. B.; Ortiz, J. V.; Cioslowski, J.; Fox, D. J.; Gaussian, Inc., Wallingford CT, 2009.

[3] (a) A. D. Becke, J. Chem. Phys. 1993, **98**, 5648. (b) C. Lee, W. Yang, R. G. Parr, Phys. Rev. B: Condens. Matter 1988, **37**, 785. (c) T. Yanai, D. Tew, N. Handy, Chem. Phys. Lett. 2004, **393**, 51. (d) R. Ditchfield, W. J. Hehre, J. A. Pople, J. Chem. Phys. 1971, **54**, 724. (e) W. J. Hehre, R. Ditchfield, Pople, J. A. Chem. Phys. 1972, **56**, 2257. (f) P. C. Hariharan, J. A. Pople, Theor. Chim. Acta 1973, **28**, 213.

[4] H. Fallah-Bagher-Shaidaei, S. S. Wannere, C. Corminboeuf, R. Puchta, P. v. R. Schleyer, Org. Lett. 2006, **8**, 863.

[5] D. Geuenich, K. Hess, F. Köhler, R. Herges, Chem. Rev. 2005,**105**, 3758.
